# Exogenous carbon monoxide improves neuronal differentiation: a near-death experience

**DOI:** 10.1186/2193-1801-4-S1-P9

**Published:** 2015-06-12

**Authors:** Ana S Almeida, Melissa Vieira, Diogo Fortunato, Helena LAVieira

**Affiliations:** CEDOC – Chronic Diseases Research Center, Portugal

**Keywords:** carbon monoxide, neuronal differentiation, neuroregeneration

Cerebral ischemic injuries and neurodegenerative disorders lead to death or impairment of neurons in the central nervous system. Application of stem cell based therapies, namely stimulation of endogenous neurogenesis or cell transplantation, are promising strategies and currently under investigation. Carbon monoxide (CO) is an endogenous product of heme degradation by heme oxygenase. Although there is no published data reporting CO as a factor involved in stem cell differentiation, several evidences support this hypothesis. This gasotransmitter induces mitochondrial biogenesis, which is also broadly described to be involved in metabolic shifts during neuronal differentiation process. Likewise, CO-induced pathways can occur via generation of small amounts of ROS, which are also important signaling molecules in neuronal differentiation. The CO effect on modulation of neuronal differentiation is assessed in three different models with increasing complexity: human neuroblastome SH-S5Y5 cell line, human teratocarcinome NT2 cell line and hippocampal organotypic slice cultures (HOSC). CO does increase the final yield of post-mitotic neurons. During neuronal differentiation, CO promotes an increase on precursor cell proliferation and in parallel CO inhibits cell death. Furthermore, cell mitochondrial population is increased by CORM-A1 supplementation. Further work is needed for assessing the mechanisms underlying CO effects in neuronal differentiation, namely by targeting modulation of cellular metabolic pathways, redox alterations and autophagy related pathways. In conclusion, CO appears as a promising therapeutic molecule to stimulate endogenous neurogenesis or to improve in vitro neuronal production for cell therapy strategies.

